# Microstructure, Thermal Conductivity, and Flame Retardancy of Konjac Glucomannan Based Aerogels

**DOI:** 10.3390/polym13020258

**Published:** 2021-01-14

**Authors:** Ying Kuang, Lijun Chen, Junjun Zhai, Si Zhao, Qinjian Xiao, Kao Wu, Dongling Qiao, Fatang Jiang

**Affiliations:** 1 Glyn O. Phillips Hydrocolloid Research Centre at HBUT, School of Food and Biological Engineering, Hubei University of Technology, Wuhan 430068, China; lazywawa@163.com (Y.K.); LijunChen202012@163.com (L.C.); Z1677169100@163.com (J.Z.); zhaosi0109@163.com (S.Z.); QJ_Xiao187166@163.com (Q.X.); wukao19@126.com (K.W.); qdttkl@163.com (D.Q.); 2 Faculty of Engineering, University of Nottingham, Nottingham NG7 2RD, UK

**Keywords:** konjac glucomannan, polysaccharide, aerogels, heat insulation, flame retardancy

## Abstract

With abundant renewable resources and good biodegradability, bio-based aerogels are considered as promising insulating materials for replacing the conventional petroleum-based foam. In this study, konjac glucomannan (KGM)-based aerogels were prepared as thermal insulation materials via a convenient sol–gel and freeze-drying progress with different content of plant polysaccharides, proteins, and wheat straw. The morphology, thermal conductivity, and flame retardancy of KGM-based aerogels were determined. The KGM-based aerogels showed a uniform three-dimensional porous microstructure. The addition of wheat straw could significantly reduce the pore size of aerogels due to its special multi-cavity structure. KGM-based aerogels showed low densities (0.0234–0.0559 g/cm^−3^), low thermal conductivities (0.04573–0.05127 W/mK), low peak heat release rate (PHRR, 46.7–165.5 W/g), and low total heat release (THR, 5.7–16.2 kJ/g). Compared to the conventional expanded polystyrene (EPS) and polyurethane (PU) foam, the maximum limiting oxygen index (LOI) of KGM-based aerogels increased by 24.09% and 47.59%, the lowest PHRR decreased by 79.37% and 94.26%, and the lowest THR decreased by 76.54% and 89.25%, respectively. The results demonstrated that the KGM-based aerogels had better performance on flame retardancy than PU and EPS, indicating high potential applications as heat insulation in the green advanced engineering field.

## 1. Introduction

With the continuous economic development, people’s living standards have been improved. The rapid increase in the skyscrapers lead to a significant increasing in energy consumption, and the carbon emissions of buildings have increased year by year [1]. To effectively slow down energy loss and reduce building carbon emissions, thermal insulation materials are commonly used as the external wall insulation layer [2]. Thermal conductivity is a very important physical index to indicate the heat insulation ability of materials [3], which refers to the heat quantity transferred along with the heat flow to the unit area of the material under the unit temperature gradient in unit time. The thermal insulation material with lower thermal conductivity had a better energy-conservation effect. In practical application, the thermal conductivity of heat insulation materials mainly depends on the chemical compositions, temperature, molecular structures, density, porosity, humidity, and other factors [4].

According to the differences in chemical composition, thermal insulation materials can be generally divided into inorganic and organic ones [5]. Typical inorganic thermal insulation materials include glass wool, asbestos, mineral wool, foam glass, etc. [6]. Organic thermal insulation materials are usually composed of synthetic or natural polymers, such as polystyrene (PS) foam [7], polyurethane (PU) foam [8,9], and biomass insulation materials [10]. Most organic materials have better thermal insulation performance than inorganic ones [11] and have advantages of low price, easy preparation, wide applicability, etc. [12]. Nevertheless, most of the synthetic polymer thermal insulation materials are unsustainable, and a large number of their wastes would take a long time to degrade after being buried. In addition, synthetic polymer materials are mainly derived from unrenewable and increasingly depleted fossil resources and aromatic groups such as toxic benzene on their chemical structures make them highly flammable. In the conflagration, synthetic polymer materials like PU may cause a fire to rapidly spread and release toxic gases, which would cause great difficulties for rescue and severely decreased the survival probability of trapped people [13,14,15]. To reduce the fire risk, various flame retardants such as halogens, phosphorus nitrogen compounds, melamine, and organoclay were added to enhance the flame retardancy of insulation materials [16,17,18,19,20]. However, some organic flame retardants have been reported to produce toxic and carcinogenic substances during combustion [21], while the application of inorganic flame retardants such as boron and silicon compounds is limited due to their weak durability and poor mechanical properties [22].

Consequently, researchers began to turn their attention to the thermal insulation materials based on natural polymers such as polysaccharides, proteins, and their derivatives [23]. In particular, natural polysaccharides have good thermal stability and mechanical properties. It has been reported that starch [24], cellulose [25], and cyclodextrin (CD) [26] are natural carbon sources, which can be used to replace conventional materials and form new environmentally friendly intumescent flame retardant systems due to the good charring ability. Furthermore, polysaccharides such as alginate and chitosan have been proved to have good retardant properties, which can significantly improve the limiting oxygen index (LOI) of thermal insulation materials [27,28].

Moreover, the chemical composition, density, and microstructure of thermal insulation materials are significant factors affecting the thermal conductivity. Ultra-light materials with high porosity, small pore size, and obturator structure usually have good thermal insulation properties [29]. Due to the advantages of large specific surface area, high porosity, low density, and low thermal conductivity, aerogel has been widely used in the thermal insulation fields since it was first invented by Kistler in 1931 [30]. At present, green and sustainable polysaccharide-based aerogels are considered to be promising thermal insulation materials instead of conventional petroleum-based foam [31,32].

Natural polysaccharides including cellulose, hemicellulose, marine polysaccharides, starch, etc. belong to the abundant renewable resources, with good safety, biodegradability, and biocompatibility [32]. Generally, the polysaccharide aerogels can be prepared by the sol-gel and the supercritical drying method. However, the supercritical drying method uses organic solvents, high pressure, and high temperature, which have potential safety hazards [33]. Due to the advantages of safety and low cost, many aerogels have been prepared by freeze-drying in recent years, e.g., chitosan and nanocellulose [34,35]. Konjac glucomannan (KGM) is a naturally abundant polysaccharide extracted from konjac, compounded of d-glucose and d-mannose connected by 1,4-glycosidic bonds with a molar ratio of 1:1.6 [36]. KGM has a high viscosity and high molecular weight and can be used as a good skeleton to form ultra-light aerogel [37,38]. According to the previous studies [39], the addition of agar, gelatin, sodium alginate, and starch could significantly improve the mechanical properties and regulate the fine pore structure of aerogels. In addition, wheat straw with continuous fibrous vascular structure was shown to have excellent thermal insulation performance [38]. Therefore, a series of KGM-based aerogels were prepared via sol–gel and freeze-drying process in this work. The microstructure, thermal insulation, and flame retardancy of KGM-based aerogels were systematically investigated and compared with the conventional thermal insulation materials such as EPS and PU. Further, the heat insulation and flame retardant mechanism of KGM-based aerogels were assumed. In summary, this work aimed to develop a green and sustainable composite aerogel with good thermal insulation and flame retardancy based on renewable biodegradable biomass polymers. This work not only proposed a new insight into preparing high-performance KGM based aerogels but also provided guidance for the design and development of environment-friendly thermal insulation materials without additive flame retardants.

## 2. Materials and Methods

### 2.1. Materials

KGM (Mw = 9.67 × 10^5^ Da) was supplied by Hubei Johnson Konjac Technology Co., Ltd. (Wuhan, China). Gelatin (ref. 10010328) was purchased from Sinopharm Chemical Reagent Co., Ltd. (Shanghai, China). Potato starch (Food grade) was purchased from Wuhan Linheji Food Co., Ltd. (Wuhan, China). Agar (Gel strength = 800–1200 g/cm^2^) was purchased from Guangzhou Saiguo Biotech Co., Ltd. (Guangdong, China). Raw wheat straw was obtained from local farmers in Wuhan and was ground into powder by a grain pulverizer and then screened through a 160 mesh sieve. Sodium alginate (Food grade) was purchased from Qingdao Bright Moon Algae Group Co., Ltd. (Qingdao, China). The EPS (Bulk density = 0.00787 g/cm^−3^) was purchased from Hubei Mesente Plastics Co., Ltd. (Hubei, China). The PU (Bulk density = 0.0539 g/cm^−3^) was purchased from Zhouning Hongshun composite Materials Business Department (Fujian China). The cylindrical mold was purchased from Thermo Fisher Scientific Co., Ltd. The conductive adhesive was purchased from Wuhan Taisheng Biotech Co., Ltd. (Wuhan, China).

### 2.2. Preparation of the KGM-Based Aerogels

KGM-based aerogels were prepared on the previously reported method [37,38] with minor modifications. For each sample, gelatin (1%, *w*/*v*), potato starch (1%, *w*/*v*), sodium alginate (1%, *w*/*v*), agar (1%, *w*/*v*), and wheat straw (1%, *w*/*v*) were, respectively, dissolved in deionized water (100 mL) in a water bath at 90 °C. Then, 1% KGM was added and mixed at a constant speed of 600 rpm for 1 h at 90 °C to obtain the mixed sol. The sol was injected into a cylindrical mold (diameter 34.8 mm, height 18 mm), the height of the sol about 10 mm, and then immediately put into a 4 °C refrigerator for age for 2 h. The aged hydrogel was placed in an ultra-low temperature refrigerator at −25 °C for 8 h, and then freeze-dried in a vacuum freeze dryer (−55 °C, 1 Pa) for 24 h to obtain the aerogel (diameter 34.8 mm, height about 10 mm). All aerogels were coded in the form of K0G0S0AL0A0WS0 (K, G, S, AL, A, WS represent konjac glucomannan, gelatin, potato starch, sodium alginate, agar, wheat straw), and the number after the K, G, S, AL, A, WS represent the weight volume percent of composition in the original sol.

### 2.3. Characterization of KGM-Based Aerogels

#### 2.3.1. Dry Density Determination

The dry density of aerogels was calculated by the following formula:

(1) $$\rho =\frac{m}{v}$$

where *m* is the weight of aerogel, and *v* is the volume of aerogel (determined by a vernier caliper).

#### 2.3.2. Scanning Electron Microscopy (SEM) and Pore Size Distribution

Before the tests, KGM-based aerogels were cut into 5 mm × 5 mm × 1 mm cubes with a blade and fixed on the stainless steel table with conductive adhesive, then coated with gold particles (Bio-Rad type SC 502, JEOL Ltd., Tokyo, Japan) for 60 s to make it conductive. Then, the microstructure of aerogels was observed by SEM (JSM6390LV, JEOL, Tokyo, Japan) at 50× and 300× magnification. The SEM images of the aerogels were loaded into Image-Pro plus 6.0 (Media Cybernetics, Inc., MD, USA) to measure its size. By manually adjusting the sensitivity to an appropriate level, the program can distinguish the contours of all pores in the images. The software can automatically find the center points of the pores and draw 180 lines through the center points at every 2°. The average value of all line segments in each pore was defined as the equivalent diameter (pore size) of the corresponding pore. Then, all the apertures were imported into EXCEL and manually counted with set intervals (10 μm) range from 10 to 240 μm. The data were drawn into a figure by Origin 2018 (Originlab, Northampton, MA, USA).

#### 2.3.3. Thermal Conductivity Determination

The thermal conductivity of KGM-based aerogels was measured at 25 °C by a Thermal Conductivity Analyzer (HOT DISK DRPL-2A, Xiangtan, China) with sensors on either side of the aerogels. The equipment was put on a stable level table with a heat shield. The temperature of the heat source sensor was controlled by a double helix thin nickel wire.

#### 2.3.4. Limiting Oxygen Index (LOI) Measurement

The LOI was measured according to ASTM D2863-97 by using a CH-2CZ oxygen index tester (Nanjing Shangyuan Analysis Instrument Company, Nanjing, China). The specimens used for the test were of dimensions 80 mm × 10 mm × 4 mm.

#### 2.3.5. Microscale Combustion Calorimeter (MCC) Measurement

The flame retardant property of KGM-based aerogels was determined by an FAA microscale combustion calorimeter (MCC, FTT0001, FTT Ltd., West Sussex, UK). About 3.0–4.0 mg of samples were placed in alumina ceramic crucible and heated between 100 and 500 °C at a heating rate of 1 °C/s in an inert nitrogen atmosphere. The decomposition products flowed from the pyrolyzer to a 900 °C combustion furnace at the 80 cm^3^/min gas stream of nitrogen and 20 cm^3^/min of oxygen, where the decomposition products were completely oxidized.

### 2.4. Statistical Analysis

All tests were performed at least in triplicate. Origin 2018 (Origin Lab Corporation, Northampton, MA, USA) was used for statistical analysis and figure drawing. SPSS (version 19, Endicott, NY, USA) was used for significant difference analysis among density and thermal conductivity of aerogels.

## 3. Results and Discussion

### 3.1. Microscopic Morphology of KGM-Based Aerogels

**Figure 1:**
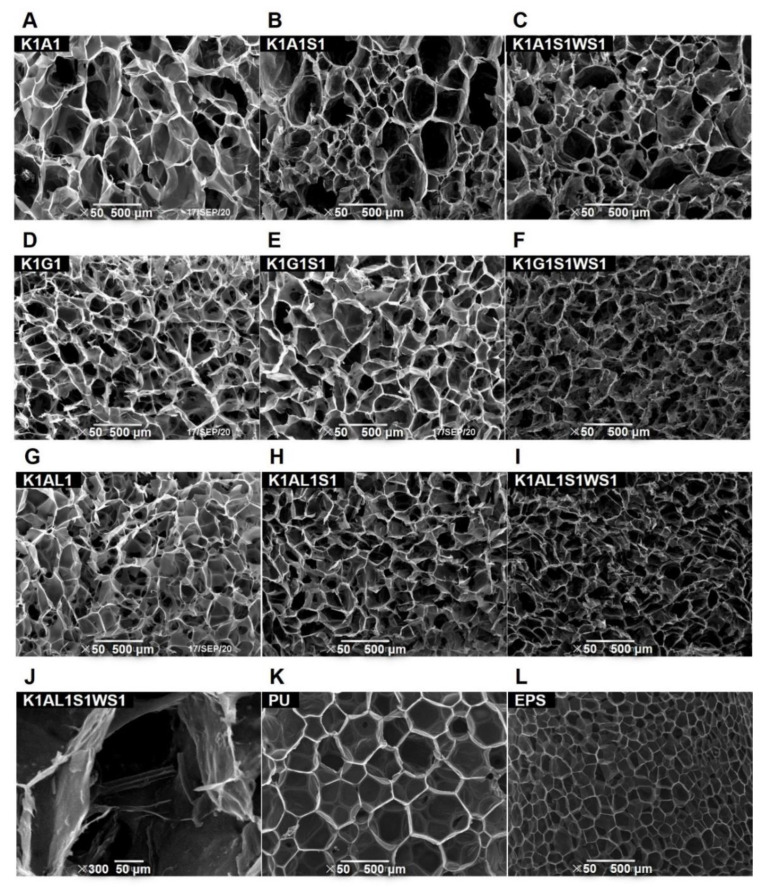
SEM images of konjac glucomannan (KGM)-based aerogels (**A**–**J**), polyurethane (PU) (**K**), and expanded polystyrene (EPS) (**L**).

**Figure 2:**
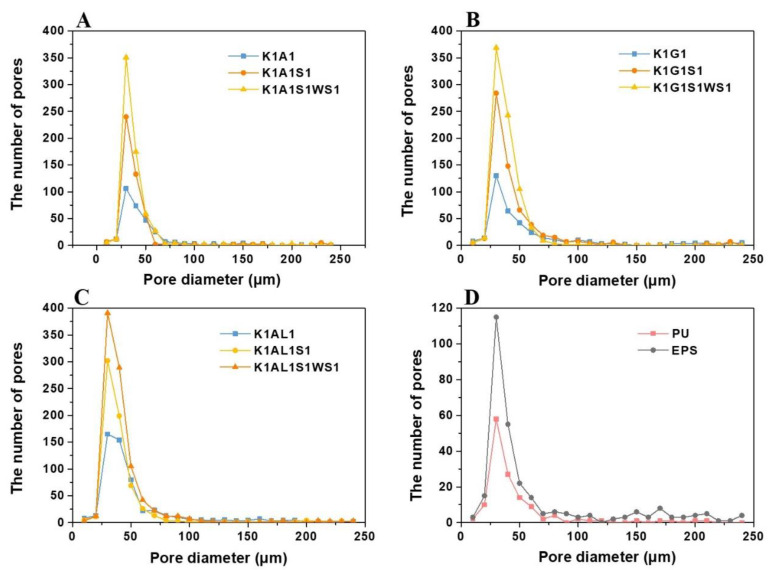
Pore size distribution of KGM-based aerogels (**A**–**C**); PU and EPS (**D**).

The surface morphology of KGM-based aerogels and conventional insulation materials (PU and EPS) were characterized by SEM (Figure 1) with the pore size distribution map shown in Figure 2. All KGM-based aerogels (Figure 1A–J) exhibited complete, uniform three-dimensional network structures, which were formed due to the sublimation of ice crystals during the freeze-drying process. It can be seen from Figure 1K,L that the PU and EPS exhibited pore shape of regular polygon. Compared with KGM-based aerogels, EPS has more closed pores and the pores were arranged in an orderly manner. With the addition of starch and wheat straw, the pore sizes of the KGM-based aerogels gradually decreased while the pore numbers of the KGM-based aerogels increased (Figure 1A–I), consistent with the results in Figure 2. Specifically, the counted total pore numbers of K1A1, K1G1, and K1AL1 aerogels were 298, 350, and 532, respectively, and the ratio of pore numbers below 50 μm of K1A1, K1G1, and K1AL1 aerogels were 82.2%, 73.7%, and 78.9%. After the addition of starch, the total pore numbers of K1A1S1, K1G1 S1, and K1AL1S1 aerogels significantly increased to 472, 622, and 651, and the ratio of pore numbers below 50 μm of K1A1S1, K1G1S1, and K1AL1S1 aerogels also increased to 92.1%, 83.0%, and 90.0%, respectively, indicating the decrease of pore sizes of KGM-based aerogels. Without wheat straw addition, most pores were round or polygonal (Figure 1A,B,D,E,G,H). After the addition of wheat straw, the pore size of aerogels (Figure 1C,F,I) became further smaller and their shapes were changed from polygons to irregular. This was explained as the addition of wheat straw changed the direction of ice crystal growth during the freezing process and, therefore, changed the shape and affected the structure and distribution of pore size of aerogels [40,41,42]. Furthermore, the wheat straw had a linear multi-cavity structure, which can bridge between the pores, making the connectivity of the porous network structure more complex (Figure 1J) and this was supported by the aperture distribution of aerogels. After the addition of wheat straw, the total pore number of K1A1S1WS1, K1G1S1WS1, and K1AL1S1WS1 aerogels further increased to 642, 783, and 911, which were about 2.2~6.7 times of those of PU and EPS. The ratio of pore numbers below 50 μm of K1A1S1WS1, K1G1S1WS1, and K1AL1S1WS1 aerogels further increased to 93.8%, 87.8%, and 94.0%, respectively, while those of PU and EPS were only 82.2% and 72.4%.

### 3.2. Thermal Conductivity of KGM-Based Aerogels

The thermal conductivity of KGM-based aerogels mainly depends on the solid thermal conductivity of the solid backbone and the gas heat conduction in the pores. Density is an important factor affecting thermal conductivity. The solid thermal conductivity of aerogel was related to the density, which varies with the concentration of the materials, and the higher the density, the higher the thermal conductivity of the solid. The density and thermal conductivity of all samples are shown in Table 1. Significant density and thermal conductivity differences were observed among different samples. K1AL1S1WS1 showed the lowest thermal conductivity (0.04573 W/mK), while K1A1 showed the highest one (0.05127 W/mK) among the samples. For K1A1, K1G1, and K1AL1, with decreasing density of aerogels, the thermal conductivity decreased slightly. After adding a small amount of starch, the density of aerogels increased slightly, but the thermal conductivity was further reduced. This may own to that the addition of a small amount of starch makes the pore walls denser, while pore size decreased, facilitates the formation of more closed pores in the aerogels and the improvement of heat insulation property [38]. After wheat straw addition, the density of KGM-based aerogels was further increased. For K1A1S1WS1, K1G1S1WS1, and K1AL1S1WS1, the thermal conductivity decreased with the decrease of density. This may own to the multi-cavity structure of the wheat straw. Linear wheat straw builds bridges between the pores (Figure 1I), and resulted in a more complicated gas flow path, leading to lower thermal conductivity. For K1A1, K1A1S1, and K1A1S1WS1, the thermal conductivity of KGM-based aerogels ranges from 0.05127 to 0.04748 W/mK, which was decreased with the density increasing. This may own to the addition of starch and wheat straw, resulting in more micron-sized closed pores in the KGM-based aerogels. Another possible explanation is that the increase of solid thermal conductivity may be less than the decrease of gaseous thermal conductivity with the increase of solid content. The density of EPS was the lowest among all the samples (0.00787 g/cm^−3^), and the thermal conductivity was the highest (0.05178 W/mK). For PU, the density was 0.0539 g/cm^−3^ and the thermal conductivity was 0.04795 W/mK. Young-Sun Jeong et al. [43] studied the thermal conductivity of different resilient materials. The thermal conductivity of both EPS and PU tended to decrease as the density increase. In this study, the density and thermal conductivity measurements of EPS and PU were consistent with the reported results.

### 3.3. Flame Retardancy of KGM-Based Aerogels

#### 3.3.1. Limiting Oxygen Index (LOI)

The LOI measurement refers to the volume fraction of O_2_ in the total gas when the material is just able to burn in the presence of only N_2_ and O_2_. LOI test is often used to determine how easily materials can burn in the air. The LOI values testing results of aerogels were listed in Table 2. For PU and EPS, the LOI values were 20.5% and 17%, respectively, both of which were lower than that of KGM-based aerogels (all >22%). The LOI of aerogels added with sodium alginate is lower than that of the other KGM-based aerogels, which may be related to the inherent incombustibility of alginate [44]. The LOI value of K1G1 was the highest among the aerogels (about 25.09%), which was 24.09% and 47.59% higher than that of Pu and EPS, respectively. The result indicated that the flame retardancy of KGM aerogels was better than that of PU and EPS.

#### 3.3.2. Heat Release Behavior

**Figure 3:**
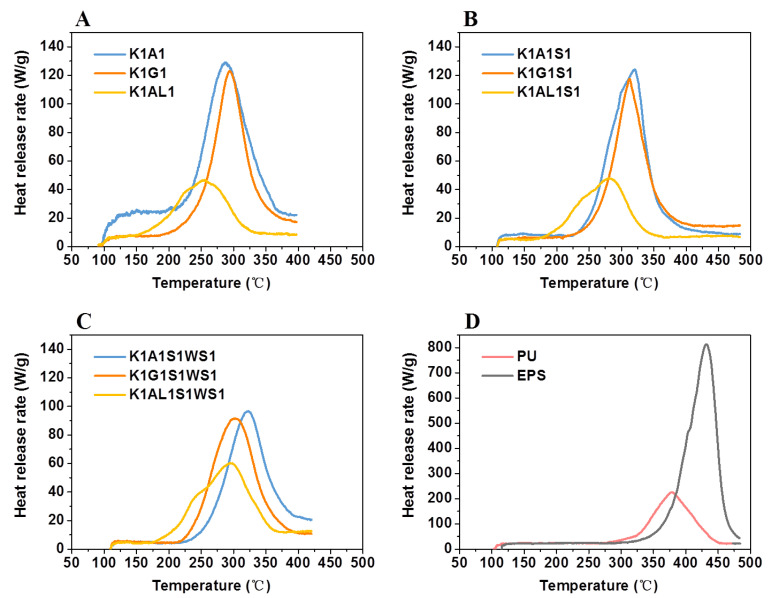
Heat release rate (HRR) curves of binary aerogels (**A**); ternary aerogels (**B**); quaternary aerogels (**C**); PU and EPS (**D**).

The microscale combustion calorimeter (MCC) was applied to analyze the flammability of the KGM-based aerogels and conventional insulation materials (EPS and PU). The HRR curves of KGM-based aerogels, EPS, and PU are shown in Figure 3 and Table 2, including the peak of heat release rate (PHRR), corresponding temperature (T_PHRR_), and total heat release (THR). The heat release rate of PU (PHRR = 226.4 W/g; T_PHRR_ = 378.2 °C) and EPS (PHRR = 813 W/g; T_PHRR_ = 431.0 °C) were significantly higher than that of KGM-based aerogels, resulted in sharp HRR curves in Figure 3D. In contrast, the sharp heat release peak of K1A1, K1G1, and K1AL1 had much smaller PHRR values of 129.2 W/g, 122.9 W/g, and 46.7 W/g, respectively. Moreover, the PHRR of the K1AL1 sample was the lowest among all samples with a corresponding temperature of 252.3 °C. After starch addition, the PHRR of KGM-based aerogels slightly decreased. Correspondingly, they were 124.1 W/g, 117.5 W/g, and 47.8 W/g, respectively. This may be attributed to the addition of starch to form more closed pores, making the oxygen needed for combustion difficult to reach the interior of the aerogels. For K1A1S1WS1 and K1G1S1WS1, due to the impact of adding wheat straw powder, the PHRRs were further reduced to 96.7 W/g and 91.7 W/g, respectively. Moreover, after adding wheat straw, the pore size of aerogels decreased significantly (Figure 1G,H), which might slow down the rate of oxygen penetration into the material. Compare with K1A1 and K1G1, the PHRR of aerogels reduced slightly after adding starch and wheat straw, while K1AL1 was the opposite. This may be that the addition of starch and wheat straw would reduce the cross-linking strength of sodium alginate in the gel process and greatly increase the density of obtained aerogels.

THR is used to measure the total heat release of material during combustion, which is determined by the flammable decomposition products. For PU and EPS samples, the THRs were 24.3 kJ/g and 53.0 kJ/g, respectively, while the corresponding values for K1A1, K1G1, and K1AL1 were 15.5 kJ/g, 10.3 kJ/g, and 6.2 kJ/g, respectively. After the addition of starch and wheat straw, THRs of the aerogels were further decreased, coincided with the PHRR of aerogels. The THR of the K1AL1S1 sample was the lowest among all samples (only 5.7 ± 0.08 kJ/g), which was 76.54% and 89.25% lower than that of PU and EPS, respectively. Moreover, the THRs of aerogels with the addition of sodium alginate were all lower than 7.5 kJ/g, which was consistent with the reported good flame-retardant effect of alginate [44]. The flame retardancy data of EPS and PU determined by microcalorimeter were different from the reported results [45,46,47], which may result from the variations of purchased samples. The PHRR and THR of all the KGM-based aerogels were much smaller than EPS and PU, indicating that the flame retardant performance of KGM-based aerogels was better than that of PU and EPS.

## 4. Conclusions

This study proposes a method to prepare aerogels with a flame retardant property using natural polysaccharides. The effects of different aerogel components on the density, microstructure, pore size distribution, heat insulation performance, and flame retardant property of KGM-based aerogels were investigated. The thermal conductivity of KGM-based aerogels was determined to be 0.04573–0.05127 W/mK, with the density of 0.0234–0.0559 g/cm^−3^, the LOI of 22.33–25.09%, the peak of heat release rate (PHRR) of 46.7–165.5 W/g, total heat release (THR) of 5.7–16.2 kJ/g, and the PHRR temperature (T_PHRR_) of 252.3–320.1 °C. The addition of starch can change the pore size and wall thickness of aerogels, and the addition of wheat straw can change the pore structure and reduce the pore size due to the multi-cavity structure of wheat straw. The PHRR and THR of KGM-based aerogels were much lower than that of conventional insulation materials such as PU and EPS. It shows that KGM aerogels had good flame retardant properties as thermal insulation. This is of great significance for the development of green flame-retardant building materials.

## Figures and Tables

**Table 1:** Density and thermal conductivity of KGM-based aerogels, PU, and EPS.

Sample	Density (g/cm^−3^)	Thermal Conductivity (W/mK)
K1A1	0.0320 ± 0.0020 ^c^	0.05127 ± 0.00124 ^c^
K1G1	0.0247 ± 0.0008 ^b^	0.04817 ± 0.00133 ^abc^
K1AL1	0.0234 ± 0.0012 ^b^	0.04705 ± 0.00120 ^ab^
K1A1S1	0.0497 ± 0.0033 ^e^	0.04980 ± 0.00156 ^bc^
K1G1S1	0.0373 ± 0.0003 ^d^	0.04795 ± 0.00163 ^abc^
K1AL1S1	0.0362 ± 0.0008 ^d^	0.04700 ± 0.00078 ^ab^
K1A1S1WS1	0.0559 ± 0.0005 ^f^	0.04748 ± 0.00156 ^ab^
K1G1S1WS1	0.0489 ± 0.0024 ^e^	0.04633 ± 0.00096 ^ab^
K1AL1S1WS1	0.0464 ± 0.0005 ^e^	0.04573 ± 0.00183 ^a^
PU	0.0539 ± 0.0021 ^f^	0.04795 ± 0.00120 ^abc^
EPS	0.00787 ± 0.00005 ^a^	0.05170 ± 0.00053 ^c^

Different superscript letters (a–f) within the same column indicate significant differences between formulations (*p* < 0.05).

**Table 2:** The flame retardancy of samples from limiting oxygen index (LOI) and microscale combustion calorimeter (MCC) tests.

Sample	LOI (%)	PHRR (W/g)	TPHRR (°C)	THR (kJ/g)
K1	<20.00	165.5 ± 1.7	320.1 ± 3.6	16.2 ± 0.6
K1A1	22.33	129.2 ± 2.1	301.5 ± 4.2	15.5 ± 0.2
K1G1	25.09	122.9 ± 1.3	313.5 ± 2.6	10.3 ± 0.2
K1AL1	24.53	46.7 ± 2.4	252.3 ± 3.2	6.2 ± 0.3
K1A1S1	22.33	124.1 ± 2.8	313.3 ± 1.7	10.9 ± 0.2
K1G1S1	24.53	117.5 ± 1.4	309.4 ± 4.1	10.0 ± 0.1
K1AL1S1	23.81	47.8 ± 2.5	280.7 ± 4.8	5.7 ± 0.08
K1A1S1WS1	22.33	96.7 ± 1.5	318.5 ± 2.4	9.2 ± 0.3
K1G1S1WS1	23.81	91.7 ± 2.3	303.5 ± 1.1	9.1 ± 0.2
K1AL1S1WS1	23.81	60.2 ± 0.9	293.9 ± 1.5	7.4 ± 0.1
PU	20.5	226.4 ± 2.0	378.2 ± 1.3	24.3 ± 0.1
EPS	17	813.0 ± 5.2	431.0 ± 3.2	53.0 ± 1.3

## Data Availability

Data available in a publicly accessible repository.
